# Radiofrequency Coblation of Congenital Nasopharyngeal Teratoma: A Novel Technique

**DOI:** 10.1155/2015/634958

**Published:** 2015-01-22

**Authors:** Sang Yun Hwang, Niall Jefferson, Alok Mohorikar, Ian Jacobson

**Affiliations:** ^1^Department of Otolaryngology and Head and Neck Surgery, Sydney Children's Hospital, Randwick, NSW 2031, Australia; ^2^Prince of Wales Hospital Clinical School, University of New South Wales, Sydney, NSW 2052, Australia

## Abstract

*Introduction*. Congenital nasopharyngeal teratomas are rare tumours that pose difficulties in diagnosis and surgical management. We report the first use of radiofrequency coblation in the management of such tumours. *Case Report*. A premature baby with a perinatal diagnosis of a large, obstructing nasooropharyngeal mass was referred to the ENT service for further investigations and management. The initial biopsy was suggestive of a neuroblastoma, but the tumour demonstrated rapid growth despite appropriate chemotherapy. In a novel use of radiofrequency coblation, the nasooropharyngeal mass was completely excised, with the final histopathology revealing a congenital nasopharyngeal teratoma. *Conclusion*. We report the first use of radiofrequency coblation to excise a congenital nasopharyngeal teratoma and discuss its advantages.

## 1. Introduction

Teratomas are congenital germ cell tumours derived from pluripotent cells from all three germinal layers [1–3]. Nasopharyngeal teratomas (NPTs) account for less than 2% of all paediatric teratomas and present unique challenges in surgical management [4].

Radiofrequency coblation technology is a relatively new surgical tool that has been adopted for use in common otorhinolaryngology procedures including adenoidectomy, tonsillectomy, and inferior turbinoplasty [5]. It has the advantages of providing haemostasis while causing minimal pain and charring of the surgical bed [6].

This case report describes the novel technique of radiofrequency coblation in the management of a congenital NPT and discusses the difficulties in diagnosis and management of such lesions.

## 2. Case History

A 40-year-old female G3 P0, with a pregnancy complicated by polyhydramnios and antepartum haemorrhage, delivered a male neonate weighing 2085 grams at 33-week gestation via an emergency Caesarean section for failure to progress through labour.

On birth, the neonate immediately developed respiratory distress requiring intermittent positive pressure ventilation (IPPV) and APGAR scores of 5 at 1 minute, 7 at 5 minutes, and 8 at 10 minutes. At 15 minutes of life, the neonate was admitted to NICU for continuous positive airway pressure (CPAP) ventilation and was intubated at 1 hour of life for worsening respiratory distress and carbon dioxide retention. The clinical course was consistent with hyaline membrane disease and a single dose of surfactant was given. Trial extubation occurred at day 1 of life.

Immediately after extubation, the neonate developed significant apnoea with desaturations. During a traumatic reintubation, a purple mass with overlying haemorrhage on the posterior pharyngeal wall, obscuring the view of the airway, was noted.

The ENT team was consulted for further investigation and management of the mass. Physical examination revealed a protruding tongue and a visible mass in the oral cavity. A laryngobronchoesophagoscopy performed on day 4 of life revealed a vascular, exophytic mass arising from the posterior pharyngeal wall, extending from the postnasal space superiorly to the level of the hypopharynx inferiorly.

**Figure 1:**
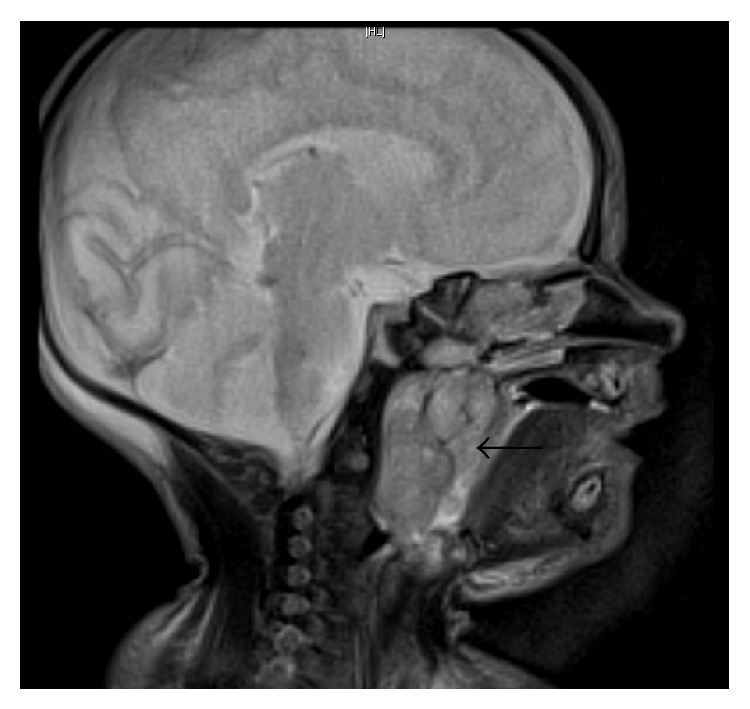
T2 weighted MRI of NPT (sagittal view).

**Figure 2:**
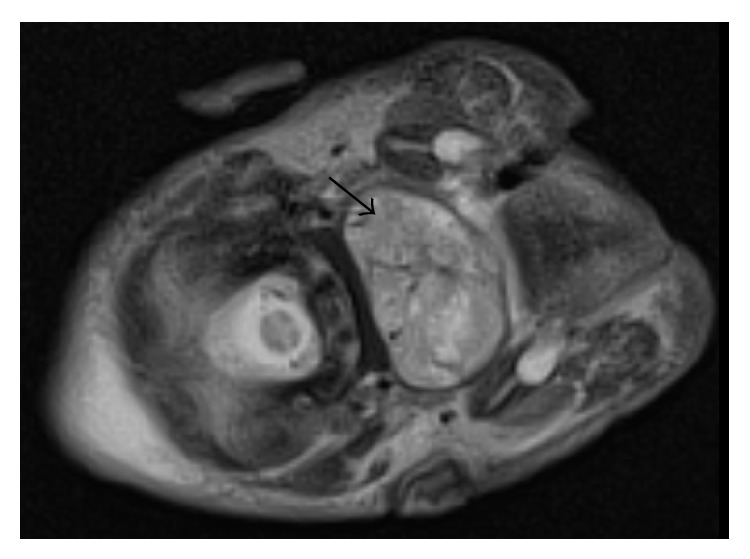
T2 weighted MRI of NPT (axial view).

A MRI was performed and demonstrated a well-circumscribed, heterogeneous mass originating from the nasopharynx, measuring 33 × 22 mm in the axial plane and 32 mm craniocaudally (Figures 1 and 2). The radiological appearance, with heterogeneous T1 and T2 signals, was suggestive of a teratoma, with differential diagnoses of rhabdomyosarcoma, haemangioma, hairy polyp, and congenital pleomorphic adenoma.

Laryngoscopy and core biopsy from the oropharyngeal component of the lesion were performed the following day. The pathology favoured a diagnosis of a differentiated neuroblastoma. As a treatment for neuroblastoma, the neonate received 1 course (3 days) of chemotherapy with carboplatin and etoposide, with prophylactic trimethoprim, sulfamethoxazole, and nystatin. However, chemotherapy-related complications of pancytopenia and febrile neutropenia developed and this was treated with G-CSF and vancomycin, piperacillin with tazobactam, and meropenem.

At this point, a clinical examination and MRI revealed an enlargement of the mass to a size of 41 × 21 × 47 mm and a second opinion of the histopathology favoured a diagnosis of teratoma.

Once the side effects of the chemotherapy were under control at 4 weeks of life, the baby underwent a perioperative tracheostomy and complete macroscopic excision of the tumour via a combined transoral and transnasal endoscopic radiofrequency coblation. A PROcise EZ View plasma wand was used to debulk the directly visualised tumour from its centre, with the overall effect of tumour collapsing in on itself.

The histopathology confirmed the diagnosis of a mostly mature NPT, with small foci immature neuroepithelium, hepatocytes, and myocytes consistent with low grade immature teratoma. No evidence of a yolk sac tumour was identified.

Decannulation of the tracheostomy was performed successfully 8 weeks postoperatively. At 18-month follow-up, the patient has no signs of recurrence and no other health concerns.

## 3. Discussion

Congenital teratomas are the most common neoplasm in a neonate, occurring in 1 in 4000 live births [1]. NPTs are much rarer, accounting for less than 2% of all paediatric teratomas [4]. There are four categories of teratoid tumours including dermoid cysts, teratoid cysts, teratomas, and epignathi [7].

The tumour in this case was a teratoma, consisting of cells from all three germ layers in varying degrees of differentiation. It exhibited mature cells, with foci of immature, poorly differentiated neuroepithelium. The latter is the likely reason for the initial biopsy being suggestive of a neuroblastoma, as the biopsy sampled an area of immature neuroepithelium, which was unrepresentative of the entire teratoma.

Based on the initial histopathological analysis, the multidisciplinary consensus was to treat the tumour as a neuroblastoma even though the radiological appearance of the tumour was most consistent with the correct diagnosis of a NPT. However, when the tumour progressed despite appropriate chemotherapy, surgical resection was organised.

In planning for surgery, radiofrequency coblation was selected as the preferred surgical technique given the vascularity of the lesion and relatively low blood volume of the 2.7 kg baby. Radiofrequency coblation uses bipolar waves to create a plasma field, which disassociates molecular bonds within soft tissues, providing haemostasis with minimal thermal damage and allowing for early wound healing [5, 8].

The tumour was debulked with radiofrequency coblation from the centre of the lesion, creating an overall effect of the tumour collapsing in on itself. This method did not require extensive dissection of tissue planes and minimised intraoperative blood loss.

However, there are limitations to the use of radiofrequency coblation in oncological procedures. Accurate histopathological assessment of tumour margins may be difficult as radiofrequency coblation leaves a 2-3 mm ablative margin around the tumour [5]. This was the case in our patient, where final histopathology suggested that the tumour was present at the margins with diathermy artefact, but clinical follow-up indicates complete resection of the tumour.

In summary, we report the first, novel use of radiofrequency coblation in the successful management of a congenital NPT.

## 4. Summary

- Nasopharyngeal teratomas are rare tumours that can cause airway obstruction.
- Diagnosis of nasopharyngeal teratomas may be difficult due to sampling error.
- Radiofrequency coblation can be used safely in excising nasopharyngeal teratomas to minimise blood loss.
- Determining microscopic margins is difficult when radiofrequency coblation is used.
